# Linac-based VMAT radiosurgery for multiple brain lesions: comparison between a conventional multi-isocenter approach and a new dedicated mono-isocenter technique

**DOI:** 10.1186/s13014-018-0985-2

**Published:** 2018-03-05

**Authors:** Ruggero Ruggieri, Stefania Naccarato, Rosario Mazzola, Francesco Ricchetti, Stefanie Corradini, Alba Fiorentino, Filippo Alongi

**Affiliations:** 1 Department of Radiation Oncology, Ospedale “Sacro Cuore – don Calabria”, Via Don A. Sempreboni 5, 37024 Negrar, VR Italy; 2 Department of Radiation Oncology, University Hospital, LMU Munich, Munich, Germany

**Keywords:** SRS, VMAT, Brain metastases, HyperArc

## Abstract

**Background:**

Linac-based stereotactic radiosurgery or fractionated stereotactic radiotherapy (SRS/FSRT) of multiple brain lesions using volumetric modulated arc therapy (VMAT) is typically performed by a multiple-isocenter approach, i.e. one isocenter per lesion, which is time-demanding for the need of independent setup verifications of each isocenter. Here, we present our initial experience with a new dedicated mono-isocenter technique with multiple non-coplanar arcs (HyperArc™, Varian Inc.) in terms of a plan comparison with a multiple-isocenter VMAT approach.

**Methods:**

From August 2017 to October 2017, 20 patients with multiple brain metastases (mean 5, range 2–10) have been treated by HyperArc in 1–3 fractions. The prescribed doses (Dp) were 18–25 Gy in single-fraction, and 21–27 Gy in three-fractions. Planning Target Volume (PTV), defined by a 2 mm isotropic margin from each lesion, had mean dimension of 9.6 cm^3^ (range 0.5–27.9 cm^3^). Mono-isocenter HyperArc VMAT plans (HA) with 5 non-coplanar 180°-arcs (couch at 0°, ±45°, ±90°) were generated and compared to multiple-isocenter VMAT plans (RA) with 2 coplanar 360°-arcs per isocenter. A dose normalization of 100%Dp at 98%PTV was adopted, while D_2%_(PTV) < 150%D_p_ was accepted. All plans had to respect the constraints on maximum dose to the brainstem (D_0.5cm3_ < 18 Gy) as well as to the optical nerves/chiasm, eyes and lenses (D_0.5cm3_ < 15 Gy). HA and RA plans were compared in terms of dose-volume metrics, by Paddick conformity (CI) and gradient (GI) index and by V_12_ and mean dose to the brain-minus-PTV, and in terms of MU and overall treatment time (OTT) per fraction. OTT was measured for HA treatments, whereas for RA plans OTT was estimated by assuming 3 min. For initial patient setup plus 5 min. For each CBCT-guided setup correction per isocenter.

**Results:**

Significant variations in favour of HA plans were computed for both target dose indexes, CI (*p* < .01) and GI (*p* < .01). The lower GI in HA plans was the likely cause of the significant reduction in V_12_ to the brain-minus-PTV (*p* = .023). Although at low doses, below 2–5 Gy, the sparing of the brain-minus-PTV was in favour of RA plans, no significant difference in terms of mean doses to the brain-minus-PTV was observed between the two groups (*p* = .31). Finally, both MU (*p* < .01) and OTT (*p* < .01) were significantly reduced by HyperArc plans.

**Conclusions:**

For linac-based SRS/FSRT of multiple brain lesions, HyperArc plans assured a higher CI and a lower GI than standard multiple-isocenter VMAT plans. This is consistent with the computed reduction in V_12_ to the brain-minus-PTV. Finally, HyperArc treatments were completed within a typical 20 min. time slot, with a significant time reduction with respect to the expected duration of multiple-isocenters VMAT.

## Background

Brain metastases (BM) are the most common intracranial tumors in adults: about 20–40% of patients affected by cancer will develop BM during their oncological history [1] and most of them have an oligometastatic disease. While multiple brain metastatic patients were typically treated by whole-brain radiotherapy (WBRT) in the past, in recent years the role of single-fraction radiosurgery (SRS) and fractionated stereotactic radiotherapy (FSRT) has gained importance in the treatment of BM [2]. This practice-changing approach has been driven by the evaluation of increased risk of detriment in neurocognitive functions for patients undergoing WBRT [3, 4], together with the absence of improvement in overall survival (OS) [5, 6] as compared to SRS/FSRT. Although SRS is now widely used for the treatment of ≤4 BM in patients with a life expectancy of more than 3–6 months [5, 7], Yamamoto et al. [8, 9] have shown similar OS for patients with 5–10 BM when compared to patients with 2–4 BM treated with SRS by Gamma Knife™ (Elekta Inc., Stockholm, Sweden). This suggests that the use of SRS/FSRT in patients with up to 10 BM might be appropriate, by limiting this upper threshold of treatable BM mainly by the duration of the treatment session [10]. Overall treatment time (OTT) remains an important issue also for linac-based treatments, which are typically performed by multiple-isocenter VMAT plans with one isocenter per lesion [11]. This translates into multiple time-consuming imaging sessions for setup correction (IGRT) corresponding to the number of isocenters. Imaging sessions are mainly performed by CBCT, which takes up to 5 min for scanning, image registration and setup correction. Therefore, an added time from IGRT to the beam-on time of about 5 min. per lesion makes linac-based SRS/FSRT unsuitable for patients with ≥5 BM. To support the use of SRS/FSRT in patients with several BM, mono-isocenter volumetric modulated arc therapy (VMAT) approaches with multiple non-coplanar arcs have been proposed [11–14], with the potential to treat multiple BM within a typical time slot (about 20 min.) thanks to the use of one IGRT session.

HyperArc™ (Varian Medical System Inc., Palo Alto, CA, USA), HA in the following, is a development of the mono-isocenter VMAT approach to SRS/FSRT first proposed by Clark et al. [11, 12], which assures a largely automated optimization process, thanks to dedicated algorithms. In August 2017, the first patients worldwide were treated with HA SRS/FSRT in our institution.

Purpose of the present analysis is to report dosimetric plan quality and OTT of single-isocenter HA plans as compared to multiple-isocenter VMAT plans.

## Methods

### Patients

From August to October 2017, 20 patients with multiple BM (mean 5, range 2–10) have received SRS/FSRT with the new HA technique in 1–3 fractions. The prescribed doses (D_p_) were as follow: 18–25 Gy in a single-fraction (if all BM had diameters smaller than 3 cm) [15]; and 21–27 Gy in three-fractions (for BM larger than 3 cm, or if located adjacent to critical structures) [16]. SRS/FSRT treatments were performed by a TrueBeam™ (Varian Inc.) linac, equipped with a ‘Millenium’ 120-leaves MLC. Although such MLC is composed of leaves of different projected widths at isocenter (0.5 cm width for the central portion of the treatment field, which is 20 cm height, and 1.0 cm width for the outer portions, 10 cm heights in each direction), only central leaves (i.e., 0.5 cm width at isocenter) were used by the here presented plans, even when (HA plans) the jaw setting was automatically selected from the system. Patients’ data are summarised in Table 1: BM originated mostly from NSCLC (8/20) and breast adenocarcinoma (7/20) as primary tumours.

**Table 1:** Patients’ gender, age, number of lesions, PTV total volume, prescribed dose and number of fractions

*Pt.#*	Gender (M/ F)	Age (y)	N. lesions	PTV (cm^3^)	D_p_ (Gy)	N. fractions
*1*	F	54	7	6.8	18	1
*2*	M	54	5	3.7	25	1
*3*	M	48	3	11.5	27	3
*4*	F	61	8	27.9	24	3
*5*	M	53	3	3.4	25	1
*6*	F	74	2	5.2	27	3
*7*	F	56	5	3.3	25	1
*8*	M	67	3	4.2	27	3
*9*	F	37	3	7.2	27	3
*10*	F	46	6	20.8	21	3
*11*	M	58	3	1.9	27	3
*12*	F	52	4	0.5	21	3
*13*	F	67	7	13.1	27	3
*14*	F	77	9	18.9	27	3
*15*	M	71	2	7.4	25	1
*16*	M	54	2	3.4	24	3
*17*	F	50	5	6.9	25	1
*18*	F	68	7	4.1	24	1
*19*	F	55	3	19.6	27	3
*20*	M	55	10	21.7	27	3
*m*		58	5	9.6	25	2
*sd*		10	2	8.0	3	1
*min*		37	2	0.5	18	1
*max*		77	10	27.9	27	3

*m*, mean; *sd*, standard deviation; *min*, minimum value; *max*, maximum value

For each patient a planning-CT scan without contrast medium (CTp) was acquired in supine position, with the Encompass™ (QFix, Avondale, PA – USA) mask and support, and a slice thickness of 1 mm. The double mask is made out of a very rigid thermoplastic material and provides anterior and posterior cranial support, thus minimizing the risk of inter/intra-fractional movements. Further, the Encompass support system includes radiopaque markers, which are used by the treatment planning system (TPS) Eclipse™ (v. 15.5.07, Varian Inc.), as described more in detail in the next section 2.2. After co-registration with the CTp, the MRI scans (3D spoiled-GRE T1) were used to contour the target volumes and organs at risk (OARs). Gross tumor volume (GTV) was defined as macroscopic contrast-enhancing lesion on T1-MRI, and was assumed equal to the clinical target volume (CTV). Planning target volume (PTV) was then obtained by an isotropic 2 mm margin from GTV, which is a common strategy to minimize risk of geographical miss, as well as to improve dosimetric accuracy, although it increases the shell of healthy brain irradiated at high doses [17, 18]. The OARs considered for the optimization were: brain (i.e., healthy brain-minus-PTV, BmP), eyes, lenses, optic chiasm, optic nerves, brainstem and hippocampi. Volumetric dose prescription was adopted, by normalizing 100% D_p_ to 98% of the volume given by the union of all the conceived PTVs (PTV_all), while large intra-tumour dose heterogeneity D_2%_(PTV) < 150%D_p_ was accepted.

### HyperArc and multiple-isocenters VMAT plans

**Fig. 1:**
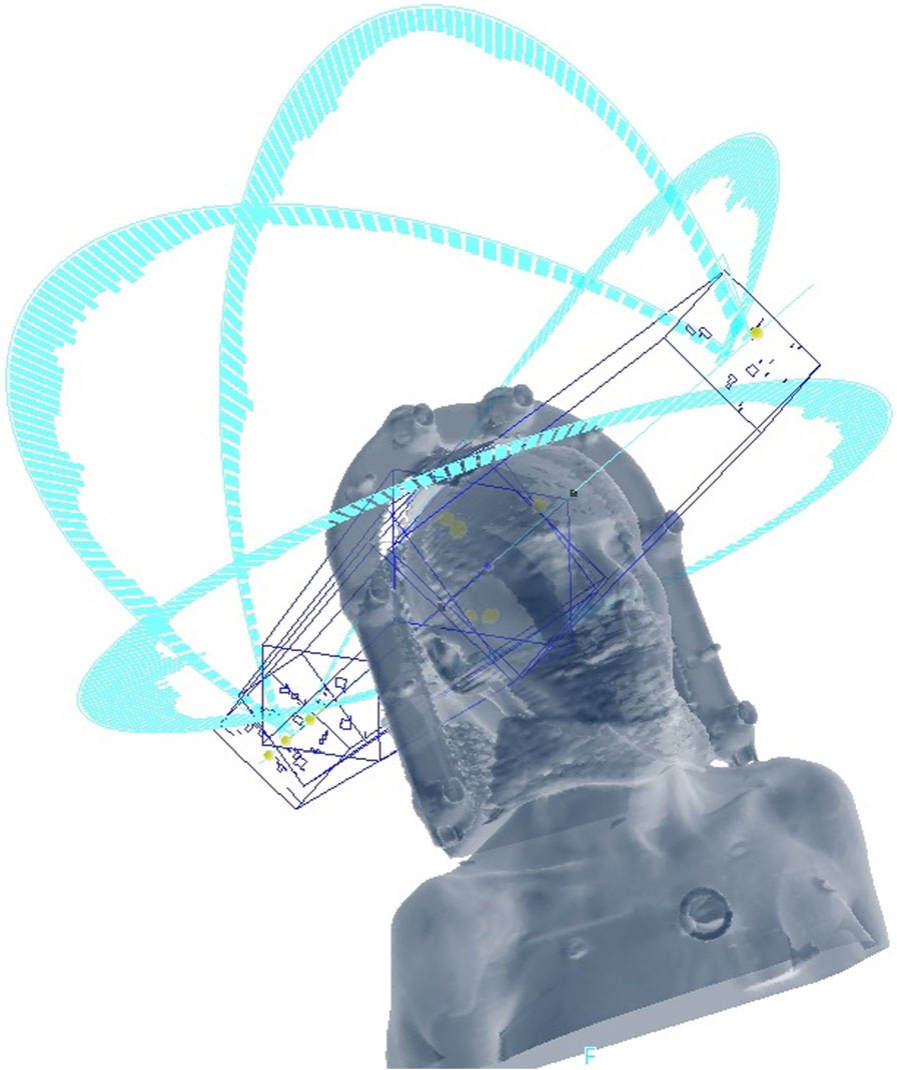
Typical beam arrangement of an HyperArc™ plan: five non-coplanar 180°-arcs at four angular positions of the couch (0°, ±45°, 90°)

HyperArc is an add-on of the Eclipse TPS which enables the use of a mono-isococentric technique for the simultaneous irradiation of multiple BM on a TrueBeam (Varian Inc.) linac. According to couch positions and arc lengths HA plans adopt the same class-solution as in [12, 13], which consists of a maximum of 5 non-coplanar arcs, based on 4 of 5 possible fixed angular couch positions to be selected between (0°, ±45°, + 90°) and (0°, ±45°, − 90°), with each arc having a fixed length of 180°, as shown in Fig. 1. HA provides a digital model of the patient support system (Encompass, Q-Fix Inc.), which enables the prediction of the clearance between the patient and the treatment machine (TrueBeam, Varian Inc.), on an individual basis, for each of the conceived non-coplanar arcs. In details, once the target volumes are specified and the planning isocenter is positioned at the barycentre of such set of targets, a preliminary test is automatically performed to estimate the position of the patient within the patient positioning device with respect to the treatment machine. This makes it possible to calculate, for the different angular positions of the couch, the distance between the patient and the treatment machine for each control point of all arcs. This strategy avoids dummy runs prior to treatment delivery at each different couch rotation and can drastically reduce the necessary OTT per treatment fraction. In case of a predicted potential collision for one of the arcs, it has to be necessarily removed first to proceed with the optimization phase. The optimization of the plan starts with the selection of the optimal collimator angle for each of the arcs, by a specific algorithm (CAO, Varian Inc.) that aims to minimize the occurrence of high-dose bridges between the different lesions. Within the framework of the optimizer (PO, Varian Inc.) used by Eclipse, two other specific algorithms were introduced: first, SRS-NTO (Varian Inc.), which reduces the dose to healthy surrounding brain tissue (brain-minus-PTV) according to a user-selectable weight, without the need of defining multiple concentric ring-structures around each target volume, as previously reported in the literature [12]. Second, ALDO (Varian Inc.), which assures that each target is covered by its prescribed dose, even in case of variable D_p_ values because HA allows for different prescribed doses to different lesions in the same patient (although in this study all plans were based on one and the same D_p_ value for all the lesions of each patient).

According to the planning approach we adopted to generate the here presented HA plans, we always started by letting CAO to automatically select the collimator angles for each arc. Then, we used the pre-defined weights which are proposed by the system for its new tools. ALDO, which allows that only lower-dose constraints be assigned to the targets, was used with a fixed weight equal 100 for each target. The fact that ALDO does not allow to limit the upper dose to the targets was not critical for our plans, because we are used to accept as large intra-tumour dose heterogeneity as D_2%_(PTV) < 150%D_p_, but such an approach might be problematic in centres were a lower intra-tumour dose heterogeneity is accepted. The same weight, 100, was used for SRS-NTO also. No other structures/objectives were included in this first trial of optimization. After dose calculation, the so resulting dose distributions were generally satisfying all our necessary constraints for planning approval. However, in the few cases where, as the result of the adjacency between a lesion and an OAR, the maximum dose constraint to the OAR had not been assured, the optimization was repeated by user control. ALDO was now unselected, by letting the user to insert one or more upper-dose constraints to the lesion/lesions, and upper-dose constraints to the involved OAR were added too. For planning approval, all plans had to respect the OAR constraints for maximum dose, referred to the hottest 0.5 cm^3^ (D_0.5cm3_). For single fraction (three-fractions) SRS, D_0.5cm3_ had to be lower than 10 (18) Gy, for the brainstem, and lower than 8 (15) Gy, for the eyes, lenses, optic chiasm, and optic nerves. Further, mean dose to the hippocampi had to be lower than 5 (7) Gy [19]. Before the treatment of each patient both a dosimetric verification in phantom and isocenter fidelity checks were performed. For individual dosimetric verification, a planar array of ionization chambers (Octavius™, PTW Inc.) was aligned first at the isocentric plane, which generally does not include any lesion and is hence useful for testing the range of medium to low doses, and then to a second plane, chosen to include the maximum number of almost coplanar lesions (at least one) and thus testing the high dose range. For isocenter fidelity checks one automatic procedure (MPC™, Varian Inc.), which is proposed by the manufacturer, was performed which tests the mechanical isocenter until maximum couch rotations (±90°) within a 10-min. time slot.

Before HA was implemented, in our center multiple BM were treated by multiple-isocenter RapidArc™ (Varian Inc.) VMAT plans (RA). Such RA plans consisted of 2 coplanar 360°-arcs per isocenter, with a fixed collimator angle (±10°) planned with Eclipse TPS. A couple of concentric rings, each 8 mm wide, were used to force the optimizer to create steep dose gradients around each lesion. In the present study, HA and RA plans were optimised for each patient, by the use of the same optimizer (PO, v.15.5.07) and dose calculation algorithm (AAA, v.15.5.07), with the same dose grid resolution (1 mm).

### Dose-volume and efficiency metrics

In terms of sparing of the brain-minus-PTV (BmP) the V_12_ (the volume receiving no less than 12 Gy) was used as a metric of plan quality, since a correlation between the extent of intermediate dose spill (e.g., V_12_) to the BmP and the risk of radionecrosis has previously been reported for cranial SRS/FSRT [20–22]. The mean dose (D_mean_) to the BmP was also analysed, as we believe that minimising the D_mean_(BmP) is advisable, regarding the higher re-treatment rates in patients with multiple BM treated with SRS.

According to target dose coverage, both the Paddick conformity index (CI), and the Paddick gradient index (GI) were used [23]. CI is defined by CI = (PTV_Dp_/ PTV)* (PTV_Dp_/ V_Dp_), where PTV_Dp_ is the fraction of PTV covered by the prescription isodose, and V_Dp_ is the prescription isodose volume (cm^3^). According to our dose normalization PTV_Dp_/ PTV = 0.98, hence CI is reduced to 0.98* (0.98* PTV/ V_Dp_). GI is defined by GI = V_50%Dp_/ V_Dp_, i.e. the ratio of the volumes delimited by the 50%D_p_ and the 100%D_p_ isodoses. The GI describes the steepness of the dose gradient from high (D_p_) to medium (50%D_p_) dose levels and is a valid surrogate to evaluate the extent of the intermediate dose spill which impacts the V_12_(BmP) and therefore the risk of radionecrosis [20–22].

The total number of monitor units (MU) and the overall treatment time (OTT) per fraction were also considered as indicators of efficiency in irradiation and in treatment time, respectively. Whereas measured for HA treatments, for RA plans OTT (min.) was estimated by assuming 3 min. for initial patient setup plus 5 min. per each CBCT-guided setup correction per isocenter.

### Statistics

HA and RA plans were compared in terms of the above-mentioned dose-volume metrics: Paddick conformity and gradient index for the target volumes and V_12_ and D_mean_ for the healthy brain tissue (BmP). The total number of monitor units and the overall treatment time per fraction, as efficiency indicators, were also compared. For each parameter, the two HA and RA samples were first tested for normality of distribution by Lilliefors test. Then, according to the results of such preliminary test, compared by a non-parametric Wilcoxon signed-rank test, or by a parametric t-test. All computations, at the 0.05 level for statistical significance, were performed by XLSTAT (v. 7.5.2, Addinsoft Inc.) add-on for Excel™ (Microsoft Inc.).

## Results

All patients were safely treated without interruption during treatment from any interlock related to the risk of a potential collision, although no dummy run was performed before the non-coplanar arcs. Dosimetric verification in phantom resulted in a passing-rate of not lower than 90%, 95%, for a γ-index computed at (2 mm, 2%), (3 mm, 3%), for all patients [24].

**Fig. 2:**
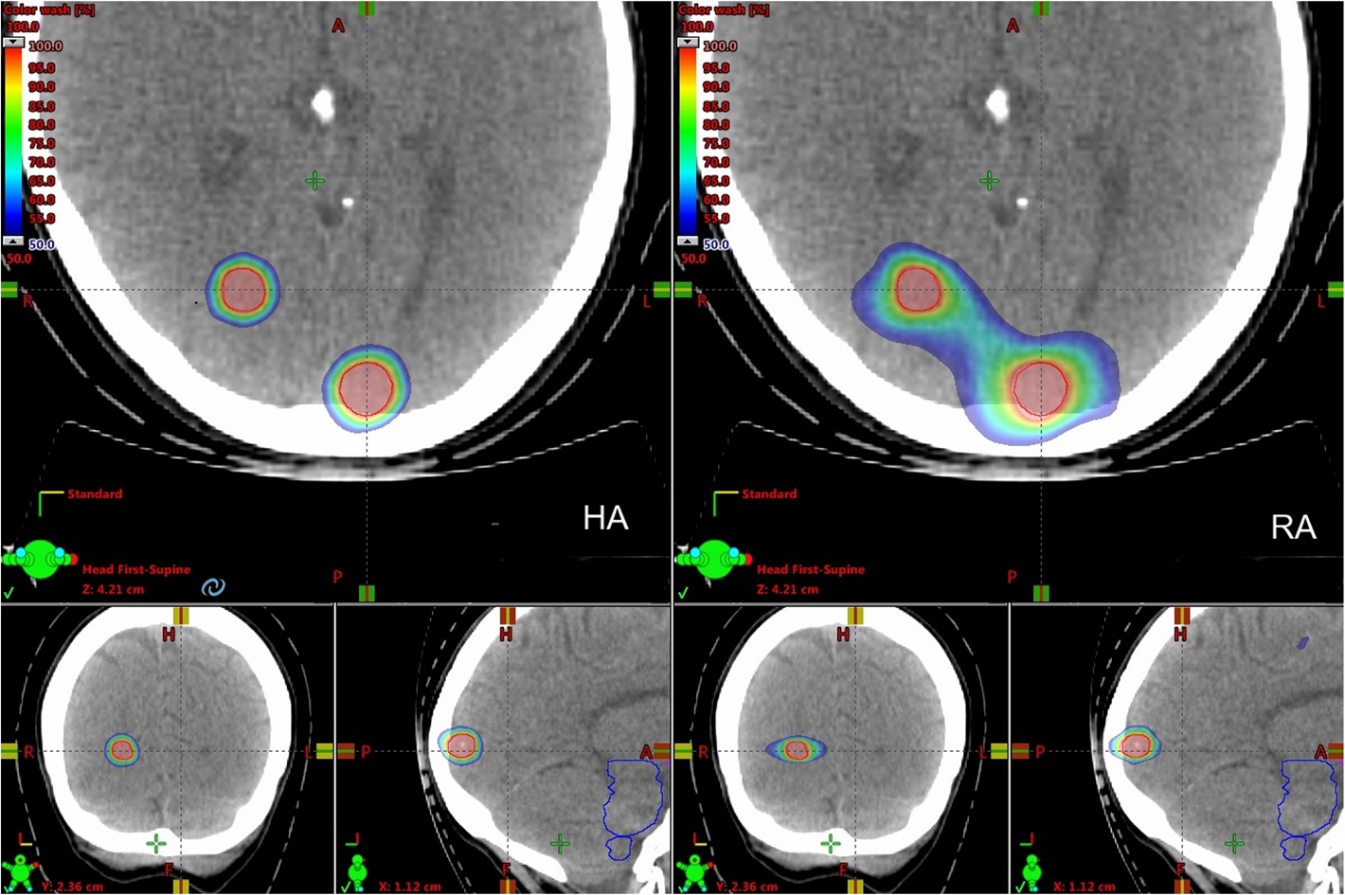
Computed dose distributions, here depicted in colourwash from 50%D_p_ to 100%D_p_, from HA (left) and RA (right) plans for an example patient. The typical enlargement of the 50%D_p_ isodose-shell around the targets for the RA plan, which may bring to the formation of dose-bridges in case of adjacent lesions, is shown

In Table 2 the values of the plan quality metrics, for both RA and HA plans, are reported for all patients. Results from hypothesis testing are reported below. For both target dose indexes significant improvements in favour of HA plans were achieved. While mean (± sd) CI value increased significantly from (0.87 ± 0.07) for RA plans, to (0.96 ± 0.02) for HA plans (*p* < .01), the GI value significantly reduced from (6.08 ± 2.24) for RA plans, to (4.41 ± 1.18) for HA plans (*p* < .01). To outline such improvement in GI from the HA plans, in Fig. 2 the spatial dose distribution between the 50%D_p_ and 100%D_p_ isodose levels is shown for a representative patient.

**Table 2:** Values of the plan quality metrics and results from hypothesis testing

	D_mean_^BmP^ (Gy)		V_12_^BmP^ (cm^3^)		CI _Paddick_		GI _Paddick_		MU (/ fraction)		OTT (min)	
*Pt.#\ Plan*	RA	HA	RA	HA	RA	HA	RA	HA	RA	HA	RA	HA
*1*	2.48	2.73	12.7	5.9	0.89	0.95	6.56	4.71	35,900	6905	57	14
*2*	2.17	2.45	21.6	8.9	0.80	0.98	6.84	4.69	32,921	7601	46	15
*3*	2.66	2.96	49.5	29.4	0.90	0.92	4.51	3.41	7643	2345	22	12
*4*	5.04	5.03	100.0	44.3	0.93	0.99	6.40	3.59	12,094	2712	50	12
*5*	1.51	1.71	15.7	7.7	0.88	0.97	6.09	4.25	22,388	6813	30	15
*6*	1.26	1.70	18.7	12.3	0.95	0.97	4.50	3.60	3947	2231	15	12
*7*	1.81	2.15	23.9	9.9	0.83	0.98	7.80	5.02	18,755	6236	38	14
*8*	1.85	2.15	23.5	13.3	0.91	0.98	5.80	4.24	6070	2421	21	12
*9*	2.49	2.83	28.1	20.4	0.92	0.94	3.82	4.61	6316	2221	21	12
*10*	3.1	4.02	34	25.1	0.93	0.93	4.15	3.36	8601	2928	38	13
*11*	1.03	1.86	12.3	6.1	0.88	1.00	6.66	4.7	6011	2778	21	13
*12*	0.66	0.75	4.8	1.7	0.72	0.96	14.3	8.85	5109	2311	26	12
*13*	4.68	4.61	66.6	31.3	0.80	0.93	5.44	3.92	14,506	3335	46	13
*14*	6.13	6.16	152.1	86.6	0.88	0.93	7.49	4.84	12,679	3842	55	13
*15*	1.86	1.82	22.9	13.9	0.93	0.96	4.69	3.49	13,069	5597	20	14
*16*	0.74	1.43	10.9	7.5	0.94	0.97	4.72	3.87	3617	2253	15	12
*17*	2.72	2.92	28.5	13.7	0.79	0.97	5.33	3.98	27,429	7843	43	15
*18*	2.32	3.00	25.9	12.0	0.69	0.94	6.30	5.01	42,737	9094	61	16
*19*	2.14	3.46	61.9	41.2	0.92	0.95	4.23	3.57	6276	2599	21	11
*20*	5.71	5.77	129.6	80.3	0.88	0.92	6.08	4.45	16,933	4197	57	13
*mean*	*2.62*	*2.98*	*42.2*	*23.6*	*0.87*	*0.96*	*6.08*	*4.41*	*15,150*	*4313*	*35*	*11*
*sd*	*1.58*	*1.47*	*40.8*	*23.6*	*0.07*	*0.02*	*2.24*	*1.18*	*11,535*	*2292*	*15*	*2*
*min*	*0.66*	*0.75*	*4.8*	*1.7*	*0.69*	*0.92*	*3.82*	*3.36*	*3617*	*2221*	*15*	*9*
*max*	*6.13*	*6.16*	*152.1*	*86.6*	*0.95*	*1.00*	*14.3*	*8.85*	*42,737*	*9094*	*61*	*15*
	*p = .310* ^*§*^		*p* =***.047***^***§***^; *p* = ***.023***^***‡***^		*p* < ***.01***^***§***^; *p* < ***.01***^***‡***^		*p* < ***.01*** ^***§***^; *p* < ***.01***^***‡***^		*p* <***.01***^***§***^; *p* < ***.01***^***‡***^		*p* < ***.01***^***§***^; *p* < ***.01***^***‡***^	

*m*, mean; *sd*, standard deviation; *min*, minimum value; *max*, maximum value. Bold characters are used for *p*-values when statistical significance resulted from 2-tails (§), or 1-tail (‡) *U* Mann-Whitney test

**Fig. 3:**
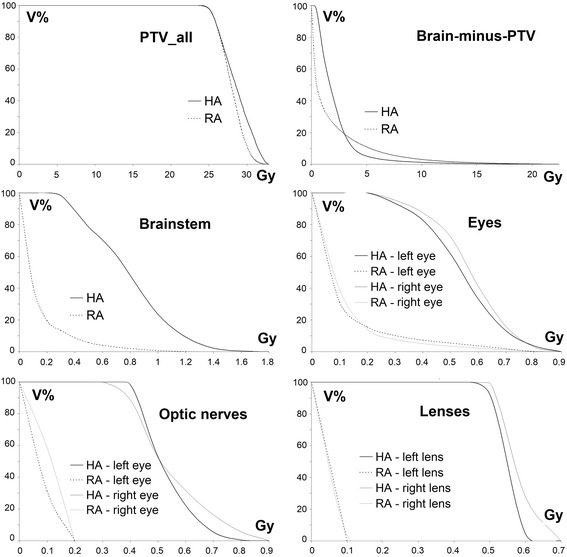
Cumulative dose volume histograms of PTV_all and OARs, from HA and RA plans, for a patient with five lesions. The typical intersection of cDVH curves for the Brain-minus-PTV is illustrated

The better GI in HA plans was most likely the cause of the significant reduction in V_12_(BmP): from (42.2 ± 40.8) cm^3^ for RA plans, to (23.6 ± 23.6) for HA plans (*p* = .023). While the results for V_12_ suggested that a better dose sparing of the BmP at the medium-to-high dose range is assured from the HA plans, the DVH curves of the BmP always exhibited an improved sparing from RA plans at low doses, typically below 2–5 Gy according to D_p_ and number of BM, as shown in Fig. 3 for a representative patient. Consistently, no significant difference between the D_mean_(BmP) of the HA, (2.98 ± 1.47) Gy, and the RA, (2.62 ± 1.58) Gy, plans were registered (*p* = .31).

According to the indicators of efficiency in irradiation and in treatment time, significant improvements were found for HA plans. The total number of monitor units per fraction significantly reduced from (15150 ± 11535) for RA plans to (4313 ± 2292) for HA plans (*p* < .01). Similarly, the overall treatment time per fraction significantly reduced from (35 ± 15) min. For RA plans to (11 ± 1) min. For HA plans (*p* < .01).

## Discussion

The present study reports the worldwide first clinical usage of the HyperArc SRS/FSRT-technique. All patients were safely treated by HA within their scheduled 20 min time slot including CBCT for setup correction. This was made possible by a digital model which includes both, the patient within the Encompass positioning setup system and the TrueBeam gantry head, and is able to predict the clearance between patient and linac for any control point of each non-coplanar arc.

To evaluate the improvements associated with the new HA technique with respect to multiple-isocenter VMAT approach (RA) to multiple BM, a comparative RA plan was generated for every patient. Noteworthy, whereas an experienced physicist is required for multi-isocenter RA plans, mono-isocenter HA plans can be generated largely automatized. The HA software selects the isocenter at the center of mass of all BM lesions, and sets the collimator angle for each arc; then, a predefined set of weighted constraints is proposed for dose optimization, which is supported by two specific algorithms (ALDO and SRS-NTO).

According to target dose coverage, the Paddick conformity index shows how conformal and overlapping the prescription isodose is with respect to the PTV. Hence, the CI value is a surrogate for the probability of local control and the ability to spare adjacent critical serial OARs. By contrast, the Paddick gradient index describes how enlarged the 50%D_p_ isodose is with respect to the prescription isodose, thus describing the extent of the medium-to-high-dose shell around the PTV.

By considering that in our sample *D*_*p*_ was equal to (25 ± 3) Gy, and thus 50%D_p_ is about equal to 12.5 Gy, a large GI directly translates into large dose spills, roughly between 12.5 and 25 Gy. This can directly be associated with an increased V_12_(BmP), which is related to the risk of radionecrosis [20–22], underlining the importance of a possibly small GI value. In cases of single-lesion SRS, an optimal value for GI of ≤3 was suggested from Paddick et al. [23]. For SRS of multiple BM, a larger upper limit for optimal GI seems still reasonable, even if the definition of an exact threshold remains challenging. In our sample the mean GI for HA plans was (4.41 ± 1.18), which is higher than 3, but still largely lower than the mean GI we obtained for RA multiple-isocenters plans (6.08 ± 2.24). Furthermore, in our preliminary experience with single-lesion HA plans we obtained a mean GI equal to (3.3 ± 0.8) (unpublished data), which is strictly consistent with the value reported from Clark et al. [12], i.e. (3.3 ± 0.4). Finally, our mean GI (4.41) is consistent with the 4.77 value reported in [25] for 3–4 BM with a range in total target size of (0.1–10.5) cm^3^. We believe this supports the high quality of plans produced by HA and that an upper threshold for optimal GI around 3.5–4.5, as a function of the number of/ distance between lesions and their absolute volume, seems reasonable for multiple BM.

According to dose sparing of healthy brain tissue, the mean value obtained for V_12_(BmP) by HA plans was almost halved when compared to RA plans ((23.6 ± 23.6) cm^3^ vs (42.2 ± 40.8) cm^3^), even if it remained still doubled with respect to the generally recommended 10 cm^3^ threshold for single lesion SRS [20–22]. However, if we restricted our computations to the 11/20 patients which had a total PTV not larger than 7 cm^3^, which reflects a mean number of 4 lesions as in [13], the mean value of V_12_(BmP) obtained with HA plans was equal to (9.0 ± 3.7) cm^3^. Further, if a per-lesion-V_12_(BmP) is computed for each patient by the ratio of V_12_(BmP) over the number of BM, similarly to Clark et al. [11], an average per-lesion-V_12_(BmP) equal to (4.9 ± 3.5) cm^3^ results which satisfies the above 10 cm^3^ threshold for single lesion SRS. Thus, HA seems well performing in terms of volume reduction for the medium-to-high dose shells around the targets.

No significant difference between HA and RA plans was computed for D_mean_(BmP), likely as the result of the increased mean paths from skin to target by the non-coplanar arcs, which holds in particular for lesions located in the posterior fossa.

A quite improved efficiency in irradiation was observed, with a mean number of MU for HA plans reduced to less than one third as compared to RA plans. This could have compensated the above-mentioned increase in the mean path from skin to target by the non-coplanar arcs, thus determining an equivalent D_mean_(BmP).

The significant reduction from HA plans in the overall treatment time per fraction (OTT) is of high clinical relevance regarding patient comfort and higher patient throughput, because with a mean OTT of (11 ± 2) min., HA treatments can be easily scheduled within a typical 20 min time slot on the treatment machine. Furthermore, HA is a fast treatment modality, independent of the number of treated BM lesions: even for patients with 5 or more lesions, for which treatments with multiple isocenters (RA) would take about 40 min or more.

## Conclusions

For linac-based SRS/FSRT of multiple brain lesions, mono-isocenter HA plans performed better than multi-isocenter VMAT plans in terms of both dose-volume quality metrics and OTT. A clinical analysis of patients’ response to treatment, focused on local control, appearance of new brain metastases and overall survival, is ongoing and will be the subject of a next report.

By considering that HyperArc produces high quality plans in a largely automated way, and enables an avoidance of collision of patient-on-couch and gantry, and that all treatments were completed within a typical 20 min. time slot, this analysis showed that HyperArc may offer an easy and safe alternative to multi-isocenter VMAT for complex SRS/FSRT for multiple BM.

## Abbreviations

BM: Brain metastases
BmP: Healthy brain-minus-PTV
CI: Paddick conformity index
CTV: Clinical target volume
Dp: Prescribed dose
DVH: Dose volume histogram
FSRT: Fractionated stereotactic radiotherapy
GI: Paddick gradient index
GTV: Gross tumor volume
HA: HyperArc™ VMAT plans
IGRT: Image guided radiotherapy
MU: Monitor units
OARs: Organs at risk
OS: Overall survival
OTT: Overall treatment time
PTV: Planning target volume
PTV_all: Union of all PTVs
RA: Multiple-isocenter VMAT plans
SRS: Single-fraction radiosurgery
TPS: Treatment planning system
V_12_: Volume receiving no less than 12 Gy
V_D_: Isodose volume
VMAT: Volumetric modulated arc therapy
WBRT: Whole-brain radiotherapy
